# Roll-to-Roll Processing of Inverted Polymer Solar Cells using Hydrated Vanadium(V)Oxide as a PEDOT:PSS Replacement

**DOI:** 10.3390/ma4010169

**Published:** 2011-01-11

**Authors:** Nieves Espinosa, Henrik Friis Dam, David M. Tanenbaum, Jens W. Andreasen, Mikkel Jørgensen, Frederik C. Krebs

**Affiliations:** 1 Department of Electronics, Computing and projects, Technical University of Cartagena, Campus Muralla del Mar. C/Doctor Fleming s/n, 30202 Cartagena, Spain; E-Mail: nieves.espinosa@upct.es (N.E.); 2 Risø National Laboratory for Sustainable Energy, Technical University of Denmark, Frederiksborgvej 399, DK-4000 Roskilde, Denmark; E-Mail: hfda@risoe.dtu.dk (H.F.D.); jewa@risoe.dtu.dk (J.W.A.); mijq@risoe.dtu.dk (M.J.); 3 Department of Physics and Astronomy, Pomona College, Claremont, CA 91711, USA; E-Mail: dtane@risoe.dtu.dk (D.M.T.)

**Keywords:** roll-to-roll printing/coating, polymer solar cells, solution processing, PEDOT:PSS free, hydrated vanadium(V)oxide

## Abstract

The use of hydrated vanadium(V)oxide as a replacement of the commonly employed hole transporting material PEDOT:PSS was explored in this work. Polymer solar cells were prepared by spin coating on glass. Polymer solar cells and modules comprising 16 serially connected cells were prepared using full roll-to-roll (R2R) processing of all layers. The devices were prepared on flexible polyethyleneterphthalate (PET) and had the structure PET/ITO/ZnO/P3HT:PCBM/V_2_O_5_·(H_2_O)_n_/Ag. The ITO and silver electrodes were processed and patterned by use of screen printing. The zinc oxide, P3HT:PCBM and vanadium(V)oxide layers were processed by slot-die coating. The hydrated vanadium(V)oxide layer was slot-die coated using an isopropanol solution of vanadyl-triisopropoxide (VTIP). Coating experiments were carried out to establish the critical thickness of the hydrated vanadium(V)oxide layer by varying the concentration of the VTIP precursor over two orders of magnitude. Hydrated vanadium(V)oxide layers were characterized by profilometry, scanning electron microscopy, energy dispersive X-ray spectroscopy, and grazing incidence wide angle X-ray scattering. The power conversion efficiency (PCE) for completed modules was up to 0.18%, in contrast to single cells where efficiencies of 0.4% were achieved. Stability tests under indoor and outdoor conditions were accomplished over three weeks on a solar tracker.

## 1. Introduction

**Figure 1:**
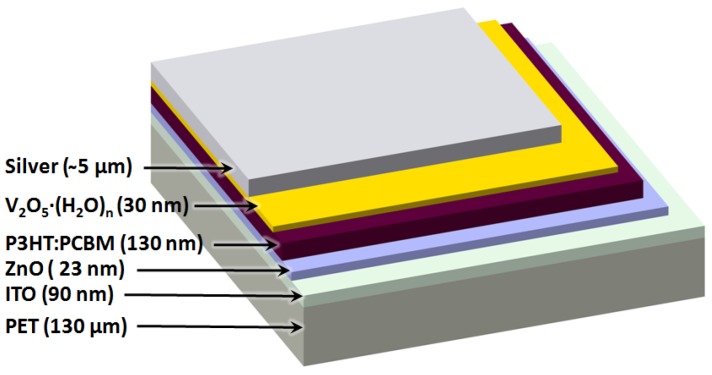
Schematic of inverted polymer solar cell structure with typical layer thicknesses shown.

Polymer solar cells [1,2,3] have seen remarkable progress in recent years and have developed from being a scientific curiosity to an emerging technology that can be manufactured industrially [4,5,6,7,8] and demonstrated in real applications [9,10,11,12,13]. Polymer solar cells have been heralded as the photovoltaic (PV) technology solving all the problems current PV technologies are faced with by providing convincing solutions to problems of cost and abundance of the materials that constitute them. The largest challenges to overcome this far have been the low performance and the short operational lifetime. Today they present power conversion efficiencies in excess of 8% [14] and estimated operational lifetimes in the range of 2–5 years [15]. The typical polymer solar cell is a multilayer structure with typically five layers stacked on top of each other. The active layer responsible for light absorption and generation of free charge carriers is typically the middle layer sandwiched between two charge selective layers, as shown in Figure 1. The two outer layers are highly conducting electrodes for extraction of the generated electrical current. One of those must be transparent. The electron selective layers have been developed recently but have otherwise been limited to the intentional use of low work function metals alone or in combination with very thin wide band gap insulators such as LiF and MgF_2_. Relatively recently, a new class of moderately conducting electron selective layers have been explored (ZnO, TiO_2_, Nb_2_O_5_) [16]. The hole selective layer has been limited almost exclusively to various formulations of PEDOT:PSS. The reasons for this are mostly historical and PEDOT:PSS was first employed as an intermediate layer that served to stabilize the work function of ITO and to planarize it, thus enabling formation of nearly defect free thin films on top [17]. PEDOT:PSS has evolved and now exist in various formulations that provide exceptionally high conductivity and transparency. In addition, PEDOT:PSS is highly stable photochemically and is stable towards oxidative conditions.

**Figure 2:**
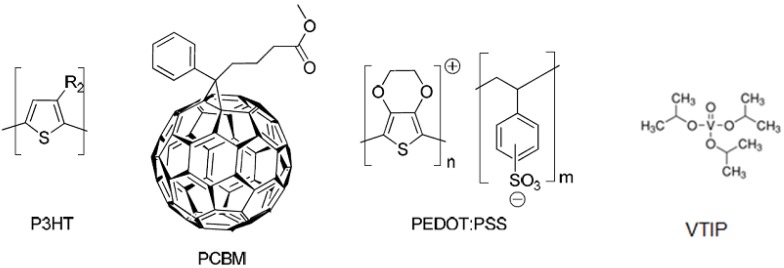
Chemical structures of materials employed in this study: P3HT, PCBM, PEDOT:PSS, and VTIP, R2 = C6H13.

The Achilles heel of PEDOT:PSS, however, is its hygroscopic nature. This problem is normally never encountered under laboratory conditions where experimenters work under relatively dry indoor conditions or in a glovebox environment with nearly no humidity. At higher humidity levels such as those encountered under outdoor conditions (>50% relative humidity) this has been found to be a detrimental and stability limiting factor. This affinity for water also represents a problem when depositing by a roll-to-roll method, due to the high surface tension of the PEDOT:PSS solutions. From this point of view, there are clear incentives to find a humidity stable alternative to PEDOT:PSS. For the purpose of this study, we constructed two different types of polymer solar cell modules: one type that was a standard device with PEDOT:PSS and another type where PEDOT:PSS had been replaced with hydrated vanadium(V)oxide. Global resources are estimated to exceed 63 million tons; making vanadium the 13th most-abundant element in the Earth's crust. The materials employed are shown in Figure 2.

**Figure 3:**
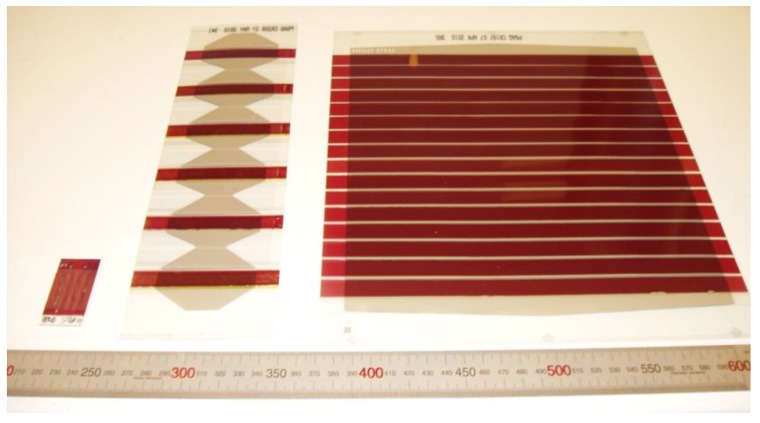
Image of a typical glass cell, gradient cell, and 16 cell module (from left to right) with a mm scale.

Binary combinations of vanadium and oxygen have a rich phase diagram with a wide range of stable compounds with different valence states for vanadium. In addition, it is common to have xerogels of these compounds with water layered between vanadium oxide sheets [18]. Processing temperatures for films on polyethyleneterphthalate (PET) substrates are limited to 140 °C. In this work, we evaluate hydrated vanadium(V)oxide as a PEDOT:PSS replacement for polymer solar cells prepared under industrially relevant conditions. We employ solutions of vanadyl-triisopropoxide (VTIP) in isopropanol and demonstrate roll-to-roll (R2R) coating of this layer in functional polymer solar cells and modules in contrast to previous OPVs [19] and OLEDs [20] where vanadium oxide films were prepared via thermal evaporation or from a spin casting powder in alcohol [21]. We further test these against a PEDOT:PSS equivalent under accelerated indoor and outdoor conditions. In this study, we have three different classes of devices, as shown in Figure 3, with active areas of 0.5, 4.2, and 360 cm^2^ for glass, gradient, and module studies, respectively.

## 2. Results and Discussion

### 2.1. Spin Coated Cells on Glass Substrates

**Figure 4:**
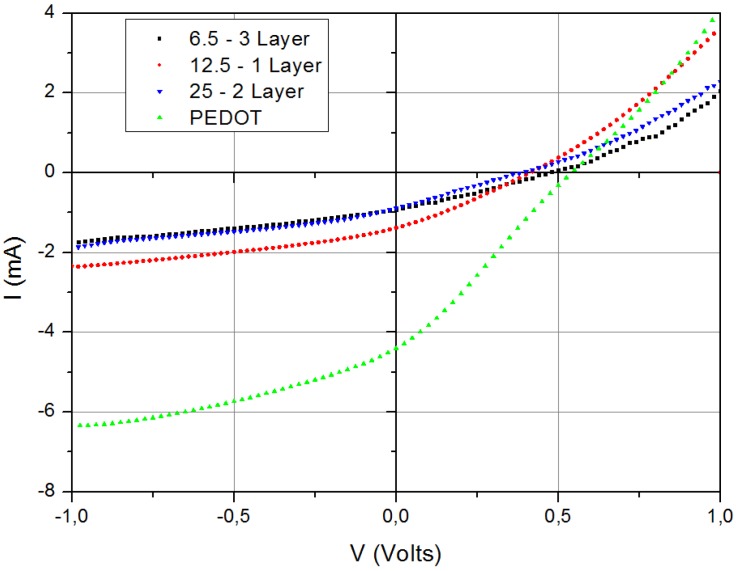
IV curves on small devices under 1,000 W/m^2^ illumination, using several VTIP concentrations, and a similar cell with PEDOT:PSS.

Small devices were prepared with a four cell substrate with ITO patterned in stripes giving 0.5 cm^2^ active area for each cell. Cells having different concentrations of VTIP in isopropanol (3.5, 6.5, 12.5, 25, 50 and 100 mg/mL), as HTL in the devices, and different numbers of layers were fabricated. Best results, shown in Figure 4, were achieved with one or two layers of 12.5 mg/mL of VTIP, yielding efficiencies around 0.4%.

These initial test results indicated that there is a range of concentration between 6.5 and 25 mg/mL for which the cells have an acceptable performance for spin coated films on glass substrates. It is clear from Figure 4 that the currents supported by the hydrated vanadium oxide films are lower than the PEDOT control sample.

### 2.2. Gradient Study on R2R PET Cells

**Figure 5:**
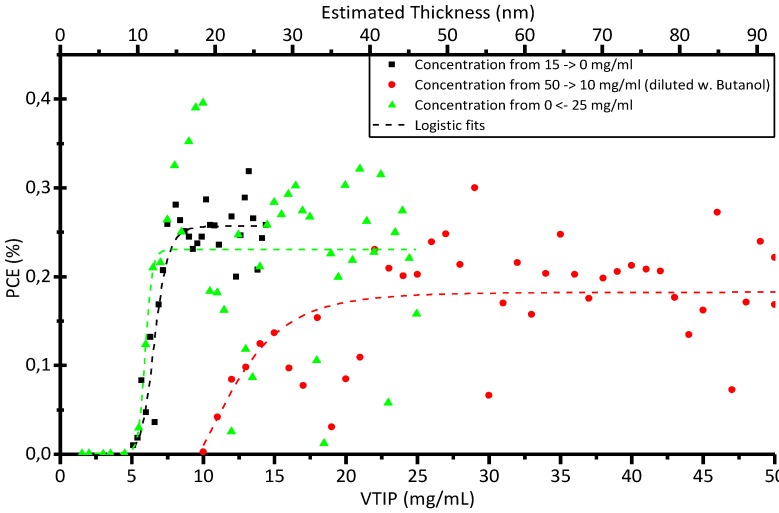
Plot of solar cell efficiency as a function of VTIP concentration in R2R gradient experiments. Estimated film thicknesses are noted on the top axis.

One critical aspect when developing new inks for R2R coating is to establish the relationship between the thickness of the dried layer that is to be coated and the coating parameters for the wet film. We have developed a method for variation of the ink properties of any layer during coating enabling identification of the optimal thickness, the critical thickness or the optimal blend ratio between donor and acceptor. In our case, we coated a gradient of the VTIP solution from zero concentration and up to 100 mg/mL. Complete devices without the hydrated vanadium oxide layer are not functional, and once the covering layer is already on top of the others, devices become functional. This is shown in Figure 5 for three series of 50 individual cells with active areas of 4.2 cm^2^.

### 2.3. Characterization of Hydrated Vanadium(V)Oxide

**Figure 6:**
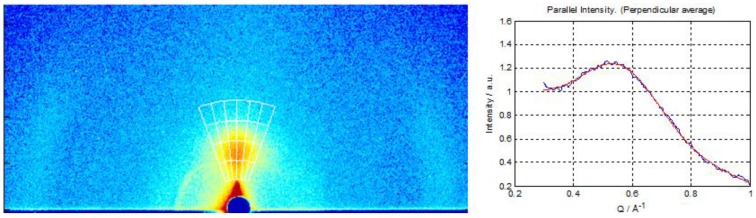
Left: The GIWAXS data as measured, with intensities represented on a color log scale. The strongest scattering feature near the center of the image corresponds to the 001 reflection, whereas the weaker scattering at the edges of the image, correspond to the 110 and 11-1 reflections [22], showing that the crystallites are preferentially oriented with the ab-plane parallel to the substrate surface. Right: Integration over 001 peak, assuming sample to detector distance of 121 mm, yields a d-spacing of 11.1 Å.

**Figure 7:**
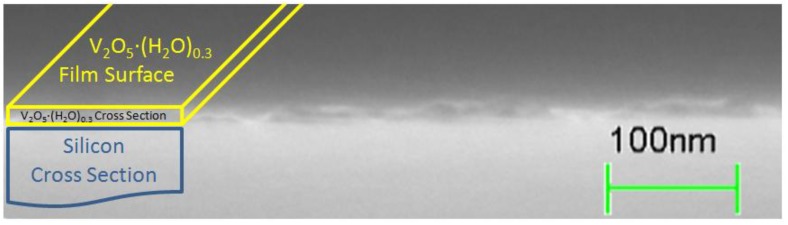
SEM cross section (tilted 17°) of 15 nm-thick cleaved V_2_O_5_·(H_2_O)_0.3_ film on a crystal silicon substrate showing film thickness and uniform morphology on the film surface.

Hydrated vanadium(V)oxide films were prepared on glass, silicon, and PET substrates and characterized by optical spectroscopy, ellipsometry, profilometry, scanning electron microscopy (SEM), energy dispersive X-ray spectroscopy (EDX), and grazing incidence wide angle X-ray scattering (GIWAXS). Measurements with GIWAXS on silicon substrates combined with the structural model of (H_2_O)_n_ [22], indicate that the films in this study are largely low-crystalline hydrated vanadium pentoxide V_2_O_5_·(H_2_O)_0.3_ with an interlayer spacing of 1.11 nm, as seen in the measurements shown in Figure 6. The hydrated vanadium pentoxide is always of low crystallinity, typically characterized as “nano-crystalline”, and the locally ordered structure was therefore determined by pair distribution function analysis [22]. EDX data confirm the relative vanadium and oxygen concentrations. A SEM image of a cleaved cross section through a 15 nm-thick film is shown in Figure 7. It is clear that the conductivity of the hydrated vanadium(V)oxide films is a limiting factor in our devices. This is a major difference between this study and previous studies where vanadium oxide films were prepared by different methods [19,21]. It has been reported that for hydrated vanadium(V)oxide films the conductivity in thicker films increases with the annealing temperature as long as the film retains the layered slab structure with a bilayer of vanadium oxide stacked between layers of water molecules. However, annealing at higher temperatures transforms the hydrated oxide to crystalline V_2_O_5_ with much lower conductivity, presumably because of the formation of grain boundaries [23]. Compared to the previously reported measurements on vanadium(V)oxide xerogel films which were cast from gel solutions [18,23], our VTIP cast films are extremely thin, with a much higher surface to volume ratio. This makes dehydration effective at much lower temperatures, so that our 120–140 °C anneal results in n values of ~0.3, comparable to much higher temperature annealing processes on the gel based films where similar n values required annealing over 250 °C.

Thickness of the roll coated films on PET were calculated using a dry film density based on this model and the known parameters of the wet coating process with Equation 1,

(1) $$t=\frac{f\cdot \rho_{w}\cdot M_{V_{2}O_{5}}}{2\cdot S\cdot W\cdot \rho_{d}\cdot M_{VTIP}}$$

where *f* is volumetric flow, *ρ_w_* and *ρ_d_* are the densities of the VTIP solution and the dry V_2_O_5_·(H_2_O)_0.3_ film, *M_V2O5_* and *M_VTIP_* are molecular weights, and *S* and *W* are the coating speed and width of the stripe.

For a PET sample coated with 15 mg/mL, a profilometer measurement of the dry film thickness showed a range with an average of 34 ± 9 nm, in agreement with the 27 nm predicted by our model.

### 2.4. Full R2R Fabrication of 16 Cell Modules

**Figure 8:**
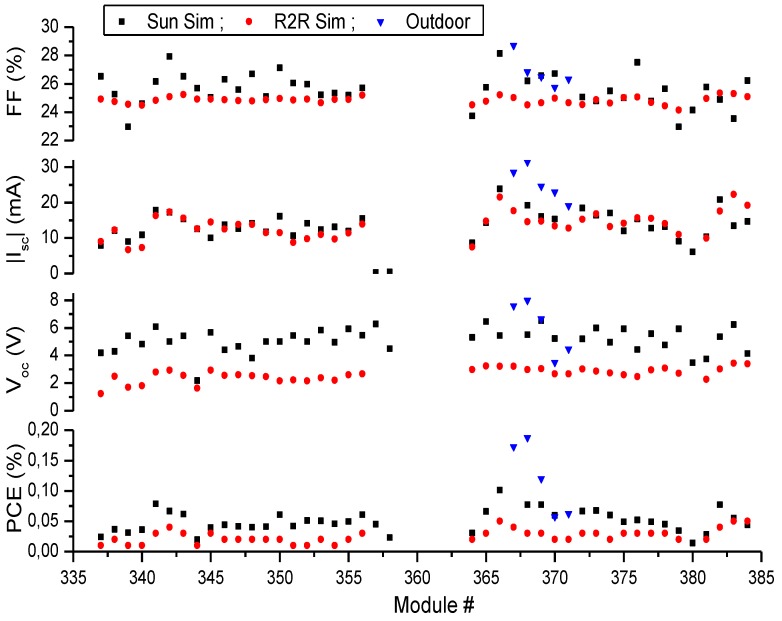
Comparison of the open-circuit voltage, *V_oc_*, the short-circuit current, *I_sc_*, the fill-factor, *FF*, and the photon conversion efficiency, *PCE*, for R2R modules made with a VTIP concentration of 15 mg/mL. They were measured under two different sun simulators and 5 modules (367–371) were measured in outdoor conditions as well.

A number of A4 size 16 cell modules with an active area of 360 cm^2^ were produced with a VTIP concentration of 15 mg/mL on PET substrates in a full R2R process. These 50 modules showed a reasonable yield, as seen in Figure 8, with a few modules around module number 360 that did not function. For the remaining modules the performance varied slightly, as seen in Table 1.

Furthermore, the use of an R2R simulator, an AM1.5G sun simulator and outdoor testing showed a significant difference between the performances of the modules. On the R2R simulator the *V_oc_* was generally lower than what was measured later on the stationary sun simulator. Interestingly, the outdoor measurements showed even greater performance with the four outdoor modules showing an increase in PCE to almost double of the indoor measurement for the modules without extra encapsulation, while the modules encapsulated in polycarbonate (PC) show a performance similar to the indoor measurements.

**Table 1:** Performance and standard deviations for the 15 mg/mL R2R modules produced and measured. The outdoor test includes both PC and PET encapsulated modules.

Measurement	# of Modules	PCE (%)	V_oc_ (V)	I_sc_ (mA)	FF (%)
**R2R Simulator**	40	0.025 ± 0.011	2.6 ± 0.5	−13.5±3.6	24.8 ± 0.3
**Sun Simulator**	40	0.050 ± 0.018	5.1 ± 0.9	−13.8 ± 3.6	25.6 ± 1.2
**Outdoor**	5	0.12 ± 0.06	6.0 ± 2.0	−25.4 ± 4.8	26.8 ± 1.1

The increased voltage obtained with the sun simulator and the outdoor measurement compared to the R2R simulator is caused by higher contact resistance in the R2R test system and by incomplete photochemical activation of the zinc oxide layer of the initially processed modules. Longer exposure (typically 20 minutes) to a high intensity of UV light in the sun simulator fully activates the zinc oxide and enhances the performance of the cells.

### 2.5. Stability Measurements

**Figure 9:**
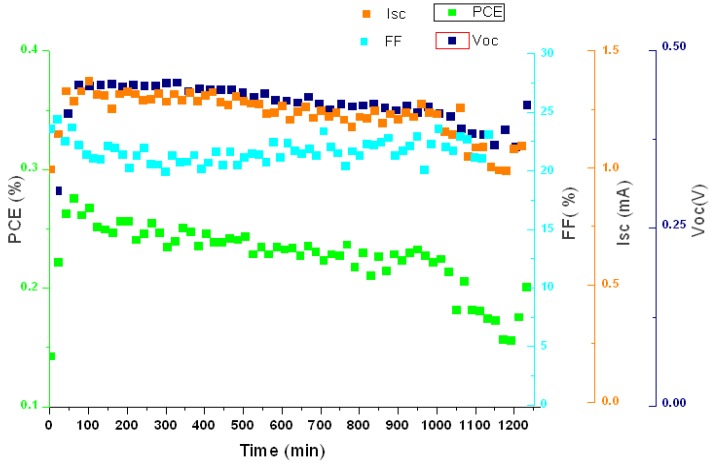
Time study over 20 hours on a glass cell with V_2_O_5_·(H_2_O)_0.3_ (25 mg/mL).

Lifetime studies were carried out under the sun simulator for all types of cells in this study. Spin coated cells on glass showed a degradation of the PCE dominated by loss of current density. The time, *T80,* for a cell to decay to 80% of its initially activated value while under AM1.5G illumination increases with film thickness, up to 18 hours for the thickest films, as shown in Figure 9. The activation of the zinc oxide layer can be seen at the beginning of the study and was similar for all the cells and modules presented here.

**Figure 10:**
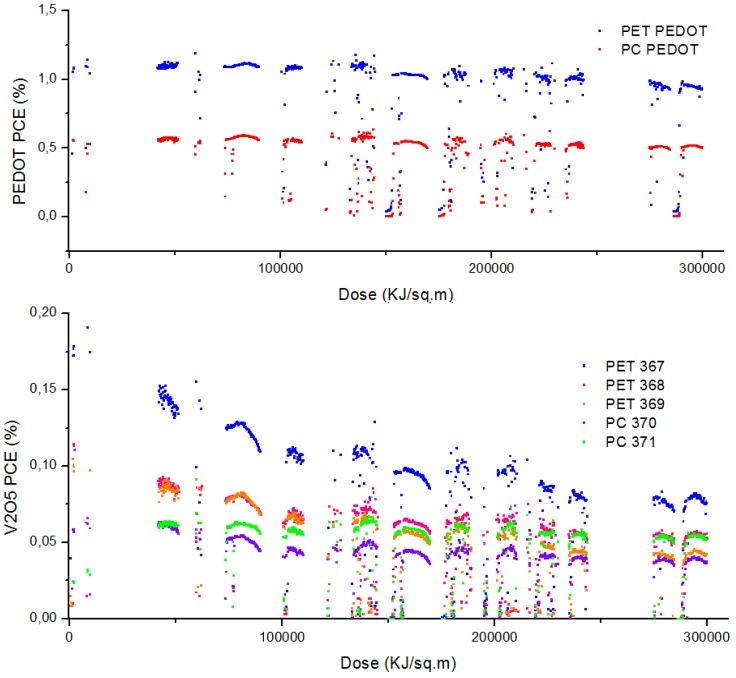

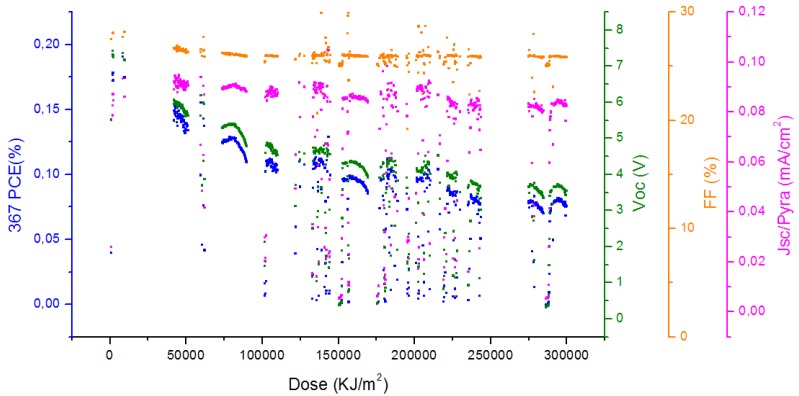
Plots of PCE for all data measured with a solar irradiance above 800 W/m^2^ plotted as a function of accumulated irradiance. The upper graph shows reference modules made with Process One using PEDOT. The middle graph shows the modules made with hydrated vanadium oxide. The full characterization, *PCE*, *V_oc_*, *FF*, and *J_sc_* (scaled by irradiance) plotted *versus* dose for module 367 is shown on the bottom graph. Modules encapsulated in polycarbonate have the prefix PC.

**Figure 11:**
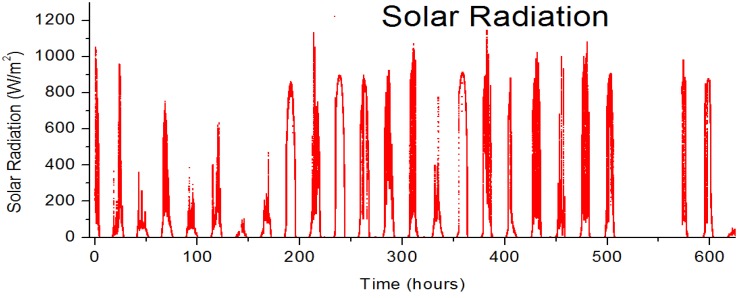
The pyranometer data on the tracking platform for the duration of the outdoor measurements reported in this study.

In contrast, cells from the gradient experiments on the R2R process had a *T80* < 30 minutes dominated by a decrease in open circuit voltage. Modules made on the R2R process were observed to have different *D80* values depending upon their encapsulation, where *D80* is the dose causing a decay to 80% of initial value. The bare PET modules having *D80~*50 MJ/m^2^ and *D50* > 300 MJ/m^2^, while two polycarbonate clad modules show *D80* > 150 MJ/m^2^ and 300 MJ/m^2^. It should be noted that modules 369 and 371 were mounted for outdoor measurements without an initial soaking in the solar simulator to photo activate the zinc oxide layer, resulting in an initial increase in performance and enhanced observed stability. Reference PEDOT modules showed minimal degradation, with *D80* > 300 MJ/m^2^, with the polycarbonate again showing a reduced efficiency with enhanced stability. The full set of performance characteristics for module 367 can be seen in Figure 10. The *J_sc_* has been scaled by the irradiance, and the dominant decay factor for the modules is the decrease in the *V_oc_* which clearly tracks the *PCE* data. The 300 MJ outdoor dose corresponds to a 28 day period with a variety of weather conditions. The pyranometer data for this period is shown in Figure 11.

## 3. Experimental Section

### 3.1. Materials

A sputtered layer of 90 nm of ITO on PET was used as the anode. ZnO nanoparticles were prepared by caustic hydrolysis of Zn(OAc)_2_·2H_2_O as described previously [4,7,9] and were used for ink formulations using Acetone as solvent. The ZnO ink was filtered through a 0.45 micron filter before coating. The ink for the active layer was prepared by dissolving P3HT (18–24 mg/mL) purchased from BASF (Sepiolid P200), and PCBM (16–22 mg/mL) purchased from Solenne B.V. in half the final volume of 1,2-dichlorobenzene at 120 °C for 3 h, followed by addition of the second half of the final volume of chloroform. The ratio between P3HT and PCBM was typically 10:9. PEDOT:PSS, was purchased as EL-P 5010 from Agfa and was diluted slowly with isopropanol using stirring until a viscosity of 270 mPa s was obtained. The hydrated vanadium(V)oxide layer was prepared by dilution of VTIP purchased from Sigma Aldrich in isopropanol. The silver ink employed, which is screen printable and heat curable was purchased from Dupont (PV 410). The adhesive for encapsulation was 467 MPF from 3M and the barrier material was purchased from Amcar and has a UV filter with a cutoff at 380 nm.

### 3.2. Grazing Incidence Wide Angle X-ray Scattering

By orienting the substrate surface just below the critical angle for total reflection with respect to the incoming X-ray beam (~0.18°), scattering from the deposited film is maximized with respect to scattering from the substrate. In the wide scattering angle range (>5°), the X-ray scattering is sensitive to crystalline structure. The GIWAXS data were acquired using a camera comprising an evacuated sample chamber with an X-ray photo-sensitive image plate with a rotating Cu-anode operating at 50 kV/200 mA as X-ray source, focused and monochromatized (Cu Kα, λ = 1.5418 Å) by a 1D multilayer [24]. Verification of the crystalline structure was done by simulating the GIWAXS pattern of the published V_2_O_5_·(H_2_O)_n_ structure [22] using the simulation software developed by Breiby *et al*. [25].

### 3.3. Processing Methods

The experiment was performed in a roll-to-roll method, following a procedure previously published and known as ProcessOne [4]. This procedure consists of slot-die coating consecutively on PET covered with ITO, zinc oxide, the active layer, and the VTIP, in a slot-die coater [4,7]. The VTIP layer was deposited in each stripe with a different gradient of concentration, starting from higher to lower, listed in Table 2. Details of the gradient coating technique are outlined in a previous publication [26]. Six gradients with 50 steps were employed in this study, comprising 300 4.2 cm^2^ cells. In addition, 50 modules with an active area of 360 cm^2^, each consisting of 16 cells in series, were produced with a fixed VTIP dilution of 15 mg/mL in isopropanol. The silver back-electrode contact was printed by means of a roll-to-roll screen printer Alraun, presented in previous publications [9]. Cells and modules were encapsulated in a barrier film with a UV filter with a cut off at 380 nm. Selected modules were further encapsulated in polycarbonate sheets with a polyurethane adhesive with an effective UV cut off at 390 nm.

**Table 2:** Summary of cells used in this study, each line represents 50 devices (400 total).

Substrate	Device	[VTIP] (mg/mL)	Active Area (cm^2^)
**Glass**	Spin Cast Single Cells	0.3–50	0.5
**PET**	R2R Cells Stripe 1	0–100	4.2
**PET**	R2R Cells Stripe 2	0–15	4.2
**PET**	R2R Cells Stripe 3	0–50	4.2
**PET**	R2R Cells Stripe 4	0–50 (butanol)	4.2
**PET**	R2R Cells Stripe 5	0–25	4.2
**PET**	R2R Cells Stripe 6	0–100	4.2
**PET**	R2R 16 Cell Modules	15	360

### 3.4. Test Conditions

All cells were measured by collecting IV curves with a Keithley 2400 sourcemeter under illumination from a solar simulator (KHS Solar Constant 1200) calibrated for AM1.5G with an automated roll to roll testing system for an initial screening, followed by an annealing soak (KHS Solar Constant 575, AM1.5G) for 20 minutes with characterization at regular intervals to activate the ZnO layer and maximize the cell performance. Modules both with and without polycarbonate encapsulation were measured outside on a solar tracking platform subjected to rain, frost, temperatures (0–13 °C), and solar radiation up to 1135 W/m^2^ at Risø DTU (Latitude: 55°41'42 N, Longitude: 12°4'16 E) Seven modules were characterized over 600 hours at 3 minute intervals throughout October 2010. The modules were open circuit between measurements (90% of the time.) Solar irradiance was recorded at 2 seconds intervals on the same tracker as the modules using a pyranometer (Eppley Lab PSP) Outdoor measurements exceed Level 1 guidelines established at the ISOS workshops [27].

## 4. Future Perspectives and Conclusion

A major perspective of this work lies in processing. Hydrated vanadium(V)oxide has been employed in the fabrication of inverted PEDOT:PSS free polymer solar cells compatible with all solution processing of all layers. The use of a coating gradient in a R2R system enables a fast and efficient way to vary device parameters. In this case, the concentration of VTIP determines hydrated vanadium(V)oxide layers of varying thickness and enables us to discern the influence this has on device performance. We observe that a minimum critical thickness for the hydrated vanadium(V)oxide layer in our process is 8 nm, beyond which device performance reaches a plateau. Overall, the performance of the devices is limited by the resistance of the hydrated vanadium(V)oxide and interface layers, which reduces the current density in comparison with optimized cells manufactured with PEDOT:PSS. Outdoor stability studies show that modules encapsulated in polycarbonate are superior to PET barrier layer materials alone.
